# Brief Report of Variants Detected in Hereditary Hearing Loss Cases in Iran over a 3-Year Period

**Published:** 2019-10

**Authors:** Niloofar BAZAZZADEGAN, Raheleh VAZEHAN, Mahsa FADAEE, Zohreh FATTAHI, Ayda ABOLHASSANI, Elham PARSIMEHR, Zahra KALHOR, Mehrshid FARAJI ZONOOZ, Fatemeh AHANGARI, Shima DEHDAHSI, Farshide SAMIEE, Payman JAMALI, Haleh HABIBI, Younes NOURIZADEH, Shokouh MAHDAVI, Maryam BEHESHTIAN, Ariana KARIMINEJAD, Richard JH SMITH, Hossein NAJMABADI

**Affiliations:** 1. Genetics Research Center, University of Social Welfare and Rehabilitation Sciences, Tehran, Iran; 2. Kariminejad-Najmabadi Pathology & Genetics Center, Tehran, Iran; 3. Genetic Medical Counseling Center, Qazvin, Iran; 4. Shahrood Genetic Counseling Center, Welfare Office, Shahrood, Iran; 5. Genetic Counseling Center, Family Health Clinic, Mobasher Hospital, Hamedan, Iran; 6. Genetic Counseling Center Welfare Organization, Ilam, Iran; 7. Welfare Institution Genetic Office, Tehran, Iran; 8. Department of Otolaryngology-Head and Neck Surgery, Molecular Otolaryngology & Renal Research Laboratories, Carver College of Medicine, University of Iowa, Iowa City, IA, USA

**Keywords:** OtoSCOPE, Hereditary hearing loss, Novel variant, Known variant

## Abstract

**Background:**

Diagnosis of hereditary hearing loss (HHL) as a heterogeneous disorder is very important especially in countries with high rates of consanguinity where the autosomal recessive pattern of inheritance is prevalent. Techniques such as next-generation sequencing, a comprehensive genetic test using targeted genomic enrichment and massively parallel sequencing (TGE + MPS), have made the diagnosis more cost-effective. The aim of this study was to determine HHL variants with comprehensive genetic testing in our country.

**Methods:**

Fifty *GJB2* negative individuals with HHL were referred to the Kariminejad-Najmabadi Pathology and Genetics Center, Tehran, one of the reference diagnostic genetic laboratories in Iran, during a 3-year period between 2014 and 2017. They were screened with the OtoSCOPE test, the targeted genomic enrichment and massively parallel sequencing (TGE + MPS) platform after a detailed history had been taken along with clinical evaluation.

**Results:**

Among 32 out of 50 *GJB2* negative patients (64%), 34 known pathogenic and novel variants were detected of which 16 (47%) were novel, identified in 10 genes of which the most prevalent were *CDH23, MYO7A* and *MYO15A.*

**Conclusion:**

These results provide a foundation from which to make appropriate recommendations for the use of comprehensive genetic testing in the evaluation of Iranian patients with hereditary hearing loss.

## Introduction

Genetic causes underlie up to 80% of prelingual hearing loss, one of the most prevalent birth defects (1). To date, more than 150 loci, i.e. about 90 genes, have been reported in non-syndromic hearing loss (http://www.hereditaryhearingloss.org).

Iran has a heterogeneous population with a high rate of consanguineous marriages (2). Such populations can be considered to be unique resources of recessive rare genetic disorders. Although HHL is not an uncommon defect, the genetic heterogeneity makes many gene-specific HL types quite rare (3). With regard to the high rate of consanguinity in Iran, which increases the risk of recurrence of autosomal recessive forms of genetic disorders such as deafness, *GJB2* mutations are the most prevalent cause of HL among several genes related to autosomal recessive nonsyndromic hearing loss (ARNSHL) (4–6). Other AR genes which are in the high prevalence category are *SLC26A4*, *MYO15A*, *MYO7A*, *CDH23*, and *PCDH15* (7).

The fact that early diagnosis in HHL cases may be helpful in prevention and treatment elucidates the need to find the most efficient and cost-effective way to investigate the genetic causes of HL in every population. Hence, this report represents our experience of investigating 50 *GJB2* negative HL cases using the OtoSCOPE test, which can screen all the genes involved in hearing loss at once.

## Materials and Methods

Fifty *GJB2* negative individuals with HHL were referred to the Kariminejad-Najmabadi Pathology and Genetics Center, Tehran, one of the reference diagnostic genetic laboratories in Iran, during a 3-year period between 2014 and 2017. Their detailed history was taken along with a clinical evaluation.

All patients completed consent forms and their family pedigrees were drawn to determine the pattern of inheritance.

Hearing thresholds were measured by pure-tone audiometry following standard protocols (8). The targeted genomic enrichment and massively parallel sequencing (TGE + MPS) platform was updated from v6 to v8 as part of our standard operating procedure, increasing the number of genes screened from 116 to 152, using custom-designed Sure Design capture technology (Agilent Technologies, Santa Clara, CA, USA). All data were filtered and analyzed using a variety of in silico mutation prediction programs including Phylop, SIFT, LRT, Mutation taster, PolyPhen (HDIV) and GERP (9). Annotated variants were also considered from the Deafness Variation Database (deafnessvariationdatabase.org).

All results were discussed at a multidisciplinary meeting. The variants in each patient were discussed individually and, in the context of unique clinical information, the most comprehensive diagnosis was provided. Positive results were confirmed via Sanger sequencing before reporting.

## Results

Overall, 50 individuals with HHL were enrolled. About 30% of probands had prelingual HL with non-syndromic phenotype. After data analysis, 34 HHL variants were detected in 32 out of the 50 individuals (Table 1) while18 did not show any hearing loss related variants. The causal variant was detected in 24 out of 33 consanguineous cases. Sixteen new variants were detected among all HL related variants in this study. Four patients had retinitis pigmentosa (RP) with HL in three causative genes, *MYO7A, CDH23* and *USH2A*, with novel and known variants. Auditory neuropathy was associated with one novel variant in the *OTOF* gene (Table 1).

**Table 1:** Detected novel and known variants in 32 patients

***Patient ID***	***Consanguinity***	***Gene***	***Nucleotide change***	***Protein change***	***Zygosity***	***Pathogenicity prediction***	***OtoSCOPE version***	***Observed features***	***Reported pheno-type(MIM#)***	***Known/No vel variant [Reference]***
D68 567	Yes	*MYO7A*	c.1708C>T	p.Arg570*	Homozygous	4/5	V6	HL	Deafness, autosomal dominant 11 Deafness [MIM# 601317]; autosomal recessive 2 [MIM#600060];	Known variant (10)
D79 212	Yes		c.1708C>T	p.Arg570*	Homozygous	4/5	V7	HL	Usher syndrome; type 1B [MIM#276900]	Known variant (10)
D79 453	No		c.5215C>T	p.Arg1739*	Homozygous	4/5	V7	HL		Known variant (11)
D72 929	Yes		c.3564_3570 del-TGCCCGG	p.Tyr1188*	Homozygous	ND	V6	HL		Known variant (12)
D78 454	Yes		c.5567delG	p.Arg1856Profs *23	Homozygous	ND	V7	HL+ CHD		Novel
D82 779	Yes		c.6028G>T	p.Asp2010Tyr	Homozygous	6/6	V7	HL+RP		Novel
D87 273	No		c.75_82delG GCGGTGG / c.3718C>T	p.Ala26Glufs*13/ p.Arg1240Trp	Heterozygous/Heterozygous	ND/5/6	V8	HL+RP		Novel/ Known
D63 292	Yes	*CDH23*	c.3491delG	p.Leu1166Trpfs *11	Homozygous	ND	V6	HL+RP	Deafness; autosomal recessive 12 [MIM#601386]; Usher syndrome; type 1D; Usher syndrome, type	Novel
D83 195	Yes		c.4562A>G	p.Asn1521Ser	Homozygous	5/6	V7	HL	1D/F digenic [MIM#601067]	Known variant (13)
D80 835	Yes		c.2897G>A	p.Arg966His	Homozygous	6/6	V7	HL		Novel
D86 014	No		c.1064C>A	p.Thr355Asn	Homozygous	5/6	V8	HL		Novel
D88 410	Yes		c.5908G>A	p.Glu1970Lys	Homozygous	4/4	V8	HL		Known variant (14)
D79 868	No	*MYO15A*	c.3956C>G	p.Ser1319Cys	Homozygous	5/5	V7	HL	Deafness, autosomal recessive 3 [MIM#600316]	Novel
D85 556	No		c.3867-1G>A/c.5810G>A	-/p.Arg1937His	Heterozygous/Heterozygous	3/4 /3/5	V8	HL		Novel/Known variant (15)
D86 357	Yes		c.9437A>C	p.His3146Pro	Homozygous	3/5	V8	HL		Novel
D81 653	Yes	*USH2A*	c.2944_2945i nsT	p.Cys982Leufs* 2	Homozygous	ND	V7	HL+RP	Usher syndrome, type 2A [MIM#276901]	Known variant (16)
D88 377	Yes		c.7501C>T	p.Gln2501*	Homozygous	4/4	V8	HL		Known variant (17)
D69 627	Yes		c.13792C>T	p.Gln4598*	Homozygous	ND	V6	HL		Novel
D86 480	Yes	*CDC14A*	c.1033C>T	p.Arg345*	Homozygous	2/4	V8	HL	Deafness, autosomal recessive 105 [MIM#616958]	Novel
D87 154	No		c.1126C>T	p.Arg376*	Homozygous	3/4	V8	HL		Known variant (18)
D75 660	Yes	*OTOF*	c.1981dupG	p.Asp661Glyfs* 2	Homozygous	ND	V6	HL	Auditory neuropathy, autosomal recessive, 1; Deafness, autosomal recessive 9 [MIM#601071]	Known variant (19)
D79 455	Yes		c.2680G>A	p.Glu894Lys	Homozygous	6/6	V7	HL		Novel
D85 222	Yes	*SLC26A4*	c.1226G>A	p.Arg409His	Homozygous	6/6	V8	HL	Deafness, autosomal recessive 4, with enlarged vestibular aqueduct [MIM#600791]; Pendredsyndrome [MIM#274600]	Known variant (20)
D87 275	Yes		c.882_883del CA	p.His294GlnfsT er35	Homozygous	ND	V8	HL		Known variant (21)
D79 301	No	*PAX3*	Deletion of exons 1–4	-	Heterozygous	ND	V7	HL+Hetero chromiairidis + White forelock	Craniofacial-deafness-hand syndrome [MIM#122880]; Waardenburg syndrome, type 1 [MIM#193500]; type 3 [MIM#148820]	Novel
D84 787	Yes	*COL11A2*	c.966dupC	p.Thr323Hisfs*1 9	Homozygous	ND	V7	HL	Deafness, autosomal recessive 53 [MIM#609706]; Deafness, autosomal dominant 13 [MIM#601868]; Otospondylomegaepiphyseal dysplasia, autosomal dominant [MIM#184840]; Otospondylomegaepiphyseal dysplasia, autosomal recessive [MIM#215150]	Novel
D68 163	No	*AIFM1*	c.1264C>T	p.Arg422Trp	Hemizygous	3/3	V6	HL	Deafness, X-linked 5 [MIM#300614]	Known variant (22)
D73 555	Yes	*TMC1*	Duplication of exons 9–12	-	Homozygous	ND	V7	HL	Deafness, autosomal recessive 7 [MIM#600974]; Deafness, autosomal dominant 36 [MIM#606705]	Novel
D88 396	Yes	*KARS*	c.1097G>C	p.Cys366Ser	Homozygous	5/6	V8	HL	Deafness, autosomal recessive 89 [MIM#613916]	Novel
D86 742	Yes	*TMPRSS3*	c.1211C>T	p.Pro404Leu	Homozygous	6/6	V8	HL	Deafness, autosomal recessive 8/10 [MIM#601072]	Known variant (23)
D88 130	Yes	*MAR-VELD2*	c.1498C>T	p.Arg500Ter	Homozygous	2/4	V8	HL	Deafness, autosomal recessive 49 [MIM#610153]	Known variant (24)
D73 519	Yes	*MITF*	c.640C>T	p.Arg214*	Heterozygous	3/4	V7	HL+White forelock	Waardenburg Syndrome type 2A [MIM#193510]; COMMAD syndrome [MIM#617306]; Tietz albinism-deafness syndrome [MIM#103500]; Waardenburg syndrome/ocular albinism, digenic [MIM#103470]	Known variant (25)

HL, Hearing loss; CHD, Congenital heart defect; RP, Retinitis pigmentosa; MIM, Mendelian Inheritance in Man; ND, Not determined

Variants with pathogenicity score were checked using a maximum of six computational methods (Phylop, SIFT, LRT, Mutation taster, PolyPhenHDIV and GERP) to study conservation of missense variants and functional significance.

## Discussion

In the present study, 50 individuals with HHL were studied. Because of the heterogeneity and the role of different loci and genes in HHL, an affordable technique was required to minimize the cost and time needed for diagnosis. OtoSCOPE was chosen for detection of causative variants related to hearing loss as it can screen all the genes involved in hearing loss at once. After evaluating with this test, 16 known variants were detected in 16 individuals in whom four showed retinitis pigmentosa (RP) and hearing loss with homozygous and compound heterozygous variants in *MYO7A*, *CDH23* and *USH2A* genes.

Twelve genes are known to cause Usher syndrome (26). In this study, only three causative genes, *MYO7A*, *CDH23* and *USH2A* with novel and known variants contributed to both RP and hearing loss. Two of these genes, *MYO7A* and *CDH23,* are among five genes involved in neuro-sensory hearing loss (26). Mutations in the *MYO15A* gene were seen in three affected individuals. Deficiency in the protein encoded by the *MYO15A* gene results in severe to profound congenital non-syndromic hearing loss (27).

Our patients with *MYO15A* gene mutations also had severe to profound phenotype. The first *MYO15A* mutations causing ARNSHL was reported in the Iranian population and believed this mutation to be a common cause of ARNSHL (28). Recently, *MYO15A* mutations accounted for 9.6% of HL in a study on 302 Iranian families affected by ARNSHL (3). In our study, 3/50 affected individuals had *MYO15A* gene mutations in which four novel and known variants were detected. This is very similar to other findings (29). One novel variant in the *USH2A* gene was detected in one individual, and one novel and one known variant were also detected in the *PAX3* and *MITF* genes, respectively, in two persons with Waardenburg syndrome and profound HL. Waardenburg syndrome is one of the most prevalent forms of autosomal dominant syndromic hearing loss (ADSHL) in Iran. It may account for 1%–4% of severe-to-profound HL (30). Recently, *PAX3* mutations were reported in a group of Iranian patients with this syndrome (31). In our study *MYO7A, CDH23, MYO15A* and *USH2A* genes were the most prevalent genes with known and novel variants. Other genes with a high rate of mutations were *CDC14A, OTOF* and *SLC26A4.*

In our clinical diagnostic laboratory, we were able to diagnose a genetic cause of deafness in 32 out of 50 persons (64%). This rate ranged from 10% to 83% in several small cohort studies (32). This perhaps reflects the higher coefficient of inbreeding in our population, as in other populations with Middle Eastern ethnicity, where the diagnostic rate is higher (72%) (33). Other patients had no hearing loss related mutations, perhaps indicating the presence of other rare causative genes identified with future whole exome or whole-genome sequencing.

## Conclusion

Platforms such as OtoSCOPE, providing comprehensive genetic screening for deafness, will allow clinicians to improve patient care by providing prognostic information, and in cases with both RP and hearing loss, offer families preventative strategies to minimize the rate of progression of retinitis pigmentosa.

## Ethical considerations

Ethical issues (Including plagiarism, informed consent, misconduct, data fabrication and/or falsification, double publication and/or submission, redundancy, etc.) have been completely observed by the authors.
